# Human Papilloma Virus-Associated Cervical Cancer and Health Disparities

**DOI:** 10.3390/cells8060622

**Published:** 2019-06-21

**Authors:** Patti Olusola, Hirendra Nath Banerjee, Julie V. Philley, Santanu Dasgupta

**Affiliations:** 1 Departments of Family Medicine, The University of Texas Health Science Center at Tyler, Tyler, TX 75708, USA; patti.olusola@uthct.edu; 2 Natural, Pharmacy and Health Sciences, Elizabeth City State University, North Carolina, Elizabeth City, NC 27909, USA; bhirendranath@ecsu.edu; 3 Medicine, The University of Texas Health Science Center at Tyler, Tyler, TX 75708, USA; julie.philley@uthct.edu

**Keywords:** cervical cancer, human papilloma virus, racial disparity, prevention, treatment

## Abstract

Cervical cancer develops through persistent infection with high-risk human papilloma virus (hrHPV) and is a leading cause of death among women worldwide and in the United States. Periodic surveillance through hrHPV and Pap smear-based testing has remarkably reduced cervical cancer incidence worldwide and in the USA. However, considerable discordance in the occurrence and outcome of cervical cancer in various populations exists. Lack of adequate health insurance appears to act as a major socioeconomic burden for obtaining cervical cancer preventive screening in a timely manner, which results in disparate cervical cancer incidence. On the other hand, cervical cancer is aggressive and often detected in advanced stages, including African American and Hispanic/Latina women. In this context, our knowledge of the underlying molecular mechanism and genetic basis behind the disparate cervical cancer outcome is limited. In this review, we shed light on our current understanding and knowledge of racially disparate outcomes in cervical cancer.

## 1. Introduction

### 1.1. Cervical Cancer Epidemiology and Risk Factors

Cervical cancer (CC) is one of the leading causes of death among women worldwide, with approximately 530,000 new cases and 275,000 deaths annually [1,2,3]. In the United States, it was estimated that 13,240 new cases of CC would be diagnosed in 2018 with an estimated death of 4170 women [4]. The major risk factors associated with CC development include high-risk human papilloma virus (hrHPV) infection, age, smoking, childbirth, use of oral contraception, and diet [1,3,5,6,7,8]. Among these various risk factors, persistent infection with hrHPV appears to be the major driver of CC development [2,3,5,8]. In the early stages, hrHPV-associated CC development is asymptomatic. The hrHPV may remain undetected if not screened in a timely manner and manifest oncogenic transformation leading to CC development [8,9].

### 1.2. Cervical Cancer Health Disparities

Although overall CC rates have decreased in the USA, significant racial health disparities exist, thus posing a challenge towards disease management [10,11,12,13]. The frequency of CC incidence remains higher among African American women compared to Caucasian American women [10,12,14,15,16]. Compared to Caucasian American women, African American women have a 60% higher incidence of CC, with increased risk of late stage diagnosis [16]. Between the years 2006 and 2010, the CC incidence in African American women was 9 per 100,000 compared to 7.2 per 100,000 in Caucasian American women [17]. The mortality rate from CC in African American women is twice that in Caucasian American women [16].

The Hispanic/Latina population is a very rapidly growing minority population in the USA, and it is estimated that by 2050, 30% of the US population will be Hispanic/Latina [17,18]. Hispanic/Latina women have the highest rate of CC, with the worst progression and a high mortality rate compared to other populations. The Hispanic/Latina women are often diagnosed with advanced stage CC and experience high mortality rates (9.5/100,000) compared to non-Hispanic/Latina women (7.5/100,000), with mortality rates that are also a little higher compared to African American women [17]. Notably, the disparate outcome in CC among African American and Hispanic/Latina women compared to the Caucasian American is unlikely to be due to the differences in screening considering the similar compliance rate in the various groups [17]. Thus, understanding the molecular biological basis of the disparate outcome in various populations is necessary.

### 1.3. Cervical Cancer Biology and Progression with HPV

**Figure 1:**
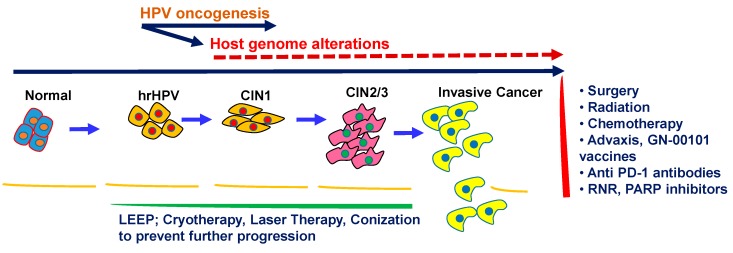
Cervical cancer development, prevention, and treatment. Invasive cervical cancer development from normal to progressive cervical intraepithelial neoplasia (CIN) through high-risk human papilloma virus (hrHPV) oncogenesis and host genome alterations. Available interventions in preventing and treating cervical cancer have also been shown. LEEP: loop electrosurgical excision procedure; RNR: ribonucleotide reductase; PARP: Poly (adenosine diphosphate [ADP]-ribose) polymerase.

**Figure 2:**
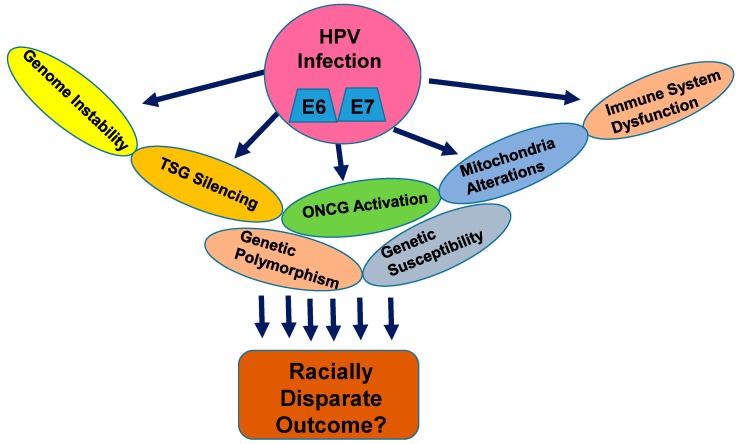
Molecular pathogenesis of HPV-associated cervical cancer. Multiple nuclear and mitochondrial genetic alteration pathways leading to cervical cancer progression and racial health disparities. E6, E7: oncogenic HPV molecules. TSG: tumor suppressor gene; ONCG: oncogene.

Cervical carcinoma arises from normal cervical epithelium through the progressive development of low grade and high grade cervical intraepithelial lesions (CINs), where hrHPV infection plays a major causative role (Figure 1). The hrHPV infection into the cervical epithelium results in host genome alterations, leading to the silencing of various tumor-suppressor factors on one hand, and inducing aberrant functioning of various tumor-promoting factors on the other. The imbalance and instability caused by various hrHPV-derived oncogenic factors into the host genome of the cervical epithelial cells drive neoplastic progression over the course of years. However, the severity of the outcomes towards CC development depends on the specific subtypes of the HPV. To date, 216 subtypes of HPV have been identified and categorized as low, medium, and high-risk types [19]. While the low and medium-risk subtypes bear low potential towards malignant transformation, the high-risk subtypes, particularly type 16 and 18, are the major promoters of neoplastic transformation. The major oncoproteins E5, E6, and E7 encoded by the HPV genome are the major drivers of oncogenesis in the normal cervical epithelium [20] and disrupt the normal functioning of the major histocompatibility complex I (MHC class I), p53 and Rb, Notch1, Wnt, MAPK, mTOR, and STAT-associated pathways, which are central players controlling normal cellular growth, differentiation, and immune function (Figure 2) [5,21]. Enhanced telomerase activity is known to be associated with epithelial cell immortalization and tumorigenesis, and hrHPV-E6 is known to activate telomerase activity in the cervical epithelium [5,21]. Thus, the oncogenic E6 and E7 components of the HPV genome have the ability to reprogram the host genome, proteome, and intracellular signaling network in the cervical epithelial niche in order to sustain and promote viral oncogenesis.

### 1.4. Nuclear Genetic Alterations in Cervical Cancer and Racial Disparities: How Far are We?

**Figure 3:**
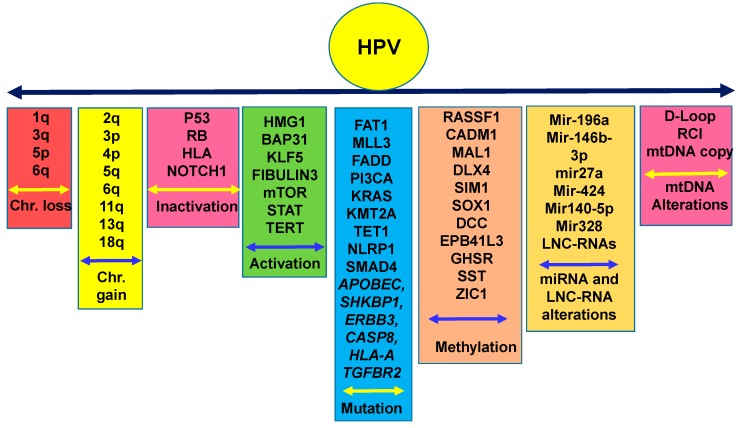
Chromosomal and molecular aberrations driving human papilloma virus-associated cervical cancer initiation, development, and progression. Frequent chromosomal copy number loss or gain along with activation and inactivation of various genes through genetic mutation, methylation, or miRNA/LNC-RNA action in cervical cancer. Chr.: Chromosome; miRNA: micro-RNA; LNC-RNA: long non-coding RNA; mtDNA: mitochondrial DNA.

To date, several molecular alterations have been documented in CC. Recent studies have identified several oncogenic factors involved in promoting CC progression, including HMGA1, BAP31, KLF5, Fibulin 3, mirRNA-196a, miR-146b-3p, and various long non-coding RNAs (Figure 3) [22,23,24,25,26,27,28]. On the other hand, the CC suppressor role of miR-27a, miR-424, mir140-5p, and mir-328 were also demonstrated [29,30,31,32]. In parallel, high throughput genome sequencing of progressive CC specimens from 120 women identified novel somatic mutations in *FAT1*, *MLL3*, *MLL2*, and *FADD* (Figure 3) [32]. In addition, this study identified HPV integration breakpoints in 97.8% of CCs, 70.5% of cervical intraepithelial neoplasias (CINs), and 42.8% of HPV^+^ normal cervical epithelium [32]. In another interesting study, exon sequencing of 409 cancer-related genes in radiation-sensitive and radiation-refractory recurrent tumors identified activating *PIK3CA* and *KRAS*, inactivating *SMAD4* mutations in the primary tumors and mutations in *KMT2A*, *TET1*, and *NLRP1* in the radiotherapy-resistant tumors [33]. In addition to these molecular alterations, chromosomal amplifications in chr.1q, 3q, 5p, 8q, and 3q26 were reported in CC (Figure 3) [6,34,35,36]. Of note, the 3q26 locus is linked to the telomerase gene, which is more frequently found to be altered in CIN lesions. A comprehensive analysis of the CC genome was also carried out through The Cancer genome Atlas (TCGA) network [37], which revealed considerable mutations in *APOBEC, SHKBP1, ERBB3, CASP8, HLA-A*, and *TGFBR2.* In addition, amplifications in closely located PD-L1 (9p24.1) and PD-L2 (9p24.1) molecules were also uncovered. Notably, the integration of high-risk HPV-18 and HPV-16 was detected in 100% and 76% cases, respectively [37]. All of these studies identified various pathways associated with CC progression. However, a connecting link between these alterations and aggressive outcome in CC health disparities is yet to be established.

### 1.5. Epigenetic Alterations in CC in Various Disparate Populations

Inactivation of tumor suppressor genes (TSGs), often achieved through promoter hypermethylation (epigenetic changes), could initiate preneoplastic and neoplastic changes in cancers including CC through progressive cervical intraepithelial neoplasia development. Epigenetic alterations are emerging as the critical determinants of cell fate in CC [38,39]. Classically, an epigenetic change will result in an addition of a methyl group on the 5-position of the cytosine (5mC) base in a CpG dinucleotide, which will eventually be oxidized into 5-hydroxymethylcytosine (5-hmc), a more stable indicator of methylation state [40]. Accumulation of these methylation signatures in CpG-rich regions around the transcriptional start site (TSS) of various genes leads to chromatin organization, which alters transcriptional activity in a locus-specific manner [40]. Considerable extents of promoter hypermethylation leading to reduced function in candidate TSGs including *P16*, *RASSF1*, *CADM1*, *MAL1*, *DLX4*, and *SIM1* were detected in CC and CIN lesions [38,39,41,42,43,44,45,46] (Figure 3). Utilizing pyrosequencing and targeted next generation bisulfite sequencing, a recent study of 167 liquid-based cytology specimens identified a three-gene methylation signature including *SOX1*, *DCC*, and *EPB41L3* in CC subjects [47] (Figure 3)**.** Employing unbiased genome-wide DNA methylation profiling and comprehensive stepwise verification and validation studies using *in vitro* and patient-derived samples, another study identified three promising methylation markers, *GHSR*, *SST*, and *ZIC1*, associated with a chromosome 3q gain for the detection of cervical preneoplasia [48]. Other than epigenetic alterations, earlier studies have also demonstrated frequent loss of chromosomal regions 2q, 3p, 4p, 5q, 6q, 11q, 13q, and 18q in CC (Figure 3) [6,34,35,36]. Thus, emerging studies have identified various molecular aberrations that could be associated with CC development and progression and may potentially be useful in biomarker and therapeutic development. However, validation of these molecules for early diagnostic/prognostic and therapeutic interventions is necessary through comprehensive analyses in various laboratories using large cohorts of clinical specimens. In addition, their utility in the context of CC health racial disparities should also be tested. 

### 1.6. Mitochondrial Genomic Alterations in CC

Reprogramming of mitochondrial (mt) dynamics and function are a hallmark of cancer, and alterations in mtDNA and their functional role in promoting tumor growth and metastases have been documented [49,50,51]. A limited number of studies have examined mtDNA alterations in CC. One study had identified numerous novel mtDNA sequence variants encompassing the non-coding D-loop as well as tRNA and rRNA genes in CC samples compared to the normal controls [52]. MtDNA sequence variants were also detected in 59% of the coding regions, with increased distribution in ND5 (respiratory complex I). A significant association between high mtDNA mutations and decreased mtDNA copy number was evident in CC [52]. Another study identified 62 sequence variants in the D-loop region of mtDNA in CC patients [53]. As opposed to the study described above, this group observed an increased mtDNA copy number in progressive cervical cancer samples compared to the corresponding normal controls [53]. In addition, an increase in reactive oxygen species (ROS) generation in CC was also noted. Other than CC, the evaluation of preneoplasic cervical lesions identified increasing mtDNA D-loop sequence variants in low grade squamous intraepithelial lesion (LGSIL, 17%) and high grade squamous intraepithelial lesion (HGSIL, 29%), whereas no mutations were detectable in the normal tissues and tissues with atypical squamous cells of undetermined significance (ASCUS) cytology [54]. Further, D-loop mtDNA mutations were detected in 67% of the CC samples [54]. This study demonstrated a progressive abundance of mtDNA alterations during CC progression from preneoplastic to neoplastic progression. 

### 1.7. Genetic Polymorphism and CC Risk

Genetic polymorphisms in various genes have been linked to the risk of development of various cancers [55]. In recent studies, polymorphic variants of various human leukocyte antigen (HLA) molecules have been linked to the development of CC [55]. Through a meta-analysis of existing data on CC, a recent study demonstrated that MspI and Ile462Val polymorphisms in *CYP1A1* gene are potential risk factors for CC development [56]. Another study suggested a potential association between *MBL2* gene exon1 polymorphisms and an increased risk of CC development [57]. Interestingly, studies of various populations in African countries, including South Africa, Zimbabwe, Morocco, Sudan, Tunisia, and Senegal, identified an association between CC risk and polymorphisms in *TGFBT10C*, *TGFBc509T*, *HLADRB1*, *CCR2V6L*, *IL-10-1082G*/*A*, and *FasR-1377G* genes [20]. However, studies are warranted to establish the connecting link between genetic polymorphisms and risk of CC in African American or Hispanic/Latina women in the USA.

### 1.8. Early Cervical Cancer Detection, Prevention, and Treatment

#### 1.8.1. Human Papilloma Virus Detection and Vaccination

The onset and development of CC is preventable through regular screening strategies using hrHPV, Pap, and colposcopy alone or in combination [1,5,7,10,11,14,18,58,59,60,61]. These tests in combination can simultaneously detect hrHPV integration and associated preneoplastic changes in the normal cervical epithelium at the very early stages. For the hrHPV screening, the Food and Drug Administration (FDA) approved cobas HPV testing [62], commonly used for women aged 25 and older. This test detects HPV types 16, 18, and 26 and additional hrHPV types. Prophylactic HPV vaccinations appear to be best choice to prevent the onset of CC. Two doses for routine HPV vaccination are now recommended for females and males aged 9–14 [63,64]. These vaccines (for example, Cervarix) are used against hrHPV subtypes 16 and 18 in the majority of cases [64]. The quadrivalent vaccine against HPV-6, 11, 16, and 18 is Gardasil; the novel nanovalent one is known as Gardasil 9, which targets HPV-6, 11, 16, 18, 31, 33, 45, 52, and 58 [64,65,66]. Of note, HPV vaccines do not offer protection for individuals with existing and stable infections. Moreover, these vaccines cannot protect against all HPV subtypes. Thus, women should be screened periodically for CC detection and follow specific and recommended guidelines.

#### 1.8.2. Pap Testing

Pap tests are liquid-based and utilize either ThinPrep or SurePath (BD Pharmingen, San Jose, CA, USA) systems and are evaluated by relevant pathologists following the guidelines of the Bethesda reporting system (BRS) [9]. Abnormalities in squamous and glandular cells are considered separately in the BRS. Atypical squamous cells (ASCs) are the most common abnormal finding in Pap tests, which is further divided into ASC-US and ASC-H [67]. The first category refers to atypical squamous cells of undetermined significance (ASC-US). These squamous cells do not appear completely normal, but there is uncertainty about the nature of the cellular changes, which could be related to hrHPV infection or other factors. The second category refers to atypical squamous cells, with a possibility of a high-grade squamous intraepithelial lesion (ASC-H). Like ASC-US, these cells also do not appear normal but could be at higher risk of being preneoplastic compared with ASC-US lesions. On the other hand, cells harboring mild dysplastic changes in the cervical epithelium caused due to HPV integration are regarded as low-grade squamous intraepithelial lesions (LGSILs). The LGSILs are also known as grade 1 cervical intraepithelial neoplasia (CIN1, Figure 1). The HPV-integrated cervical epithelial cells with more pronounced changes compared to CINI are regarded as high-grade squamous intraepithelial lesions (HGSILs) or CIN2, CIN2/3, or CIN3 depending on the degree of severity of the pathologic changes. The HGSILs are more likely to progress to carcinoma in situ (CIS) or CC if left untreated.

#### 1.8.3. Colposcopic Testing

Colposcopy is generally performed using illumination and magnification after applying 5% acetic acid for women with abnormal pap/HPV outcomes [10]. In addition to cervical biopsies, endocervical curettages are also performed in certain clinical situations, including an unsatisfactory colposcopy following low-grade intraepithelial lesion, colposcopy evaluation of high-grade squamous intraepithelial lesion, and evaluation of all subcategories of atypical glandular cell cytology. Women positive for hrHPV-16 or hrHPV-18 should undergo colposcopy examination. On the contrary, women negative for hrHPV-16 and hrHPV-18 but positive for one of the 12 other hrHPVs should undergo a Pap test to determine whether a colposcopy is necessary. Women diagnosed with CIN-2 or more advanced lesions would receive further treatment depending on age, pregnancy status, and fertility situation. The treatment options include loop electrosurgical excision procedure (LEEP), cryotherapy (low grade CINs), laser therapy, and conization (Figure 1) [9,10,17,58,68].

#### 1.8.4. Treatment for Cervical Cancer and Therapeutic Vaccines

If not screened periodically followed by preventive treatment in the pre-cancer stages, cervical cancer may develop eventually. In early stages of CC, surgery is the treatment choice. The standard of care in most progressive CC involves systemic platinum-based chemotherapy and radiotherapy in combination (Figure 1) [2,5,21,33,69]. Immunomodulatory vaccination is another choice for effectively treating hrHPV-integrated CC subjects, which can be used alone or in combination with chemoradiation therapy. Advaxis (ADXS11-011, Advaxis Inc., NJ, USA) is a unique immunotherapy vaccine. It is generated in a gram-positive bacterium *Listeria monocytogenes* and engineered to express HPV-16-E7) and has shown promising therapeutic efficacy [70] (Figure 1). GN-00101 is another therapeutic vaccine harboring *Mycobacterium bovis* heat shock protein (Hsp65) covalently linked to an entire HPV16-E7 sequence [71,72]. This vaccine has elicited anti-tumor response and also demonstrated activity against CIN lesions. In addition, promising outcomes from treatment of CC subjects with anti-PD1 antibodies, ribonucleotide reductase (RNR), and Poly (adenosine diphosphate [ADP]-ribose) polymerase (PARP) inhibitors have been reported [73,74,75].

### 1.9. The Road Ahead 

#### 1.9.1. Screening and Preventive Strategies to Reduce Disparate Outcome

The incidence of CC and associated mortality has been reduced appreciably with the implementation of hrHPV and Pap test-based screening strategies. However, significant occurrence of CC still remains a threat in certain populations, such as Hispanic/Latina and African American women, particularly in rural communities [14,15,60,76,77]. This disproportionate burden of CC in Hispanic/Latina and African American women may be partly attributable to a lack of adequate health insurance, which prevents them from receiving the periodic CC preventive screening and follow up [3,7,9,12,13,14,16,17,18,59,76,78,79]. This socioeconomic stress factor puts these women at higher risk for CC development in their life time. Considering the high incidence, late diagnosis, and mortality rate among the Hispanic/Latina women, a more rigorous CC screening approach should be employed. Adequate measures and initiatives should be taken in a timely manner to screen these underserved women to prevent CC onset.

#### 1.9.2. The Molecular Biological Basis of CC Racial Health Disparities

Numerous studies as described above and depicted in Figure 2 and Figure 3 have identified different types of nuclear and mitochondrial genetic alterations in CC in general. However, the identification and characterization of the molecular biological pathways distinctively driving aggressive outcomes in racially disparate populations and leading to higher mortality have yet to be established. Future studies in these directions are warranted to develop clinically applicable preventive and therapeutic strategies for better CC management as are being developed for various other cancers in this era of precision medicine [80,81,82]. With the advent of cutting-edge technologies including next-generation deep sequencing, transcriptome, methylome, kinome, whole genome, proteome profiling, and genome editing tools, the next decade will likely advance our knowledge in the field and lead to the development of better management strategies to reduce the gap in CC racial health disparities.

## 2. Conclusions

Although decades of research have reduced overall CC incidence, not all sections of society have equally benefitted. Indeed, certain ethnic and racial populations, particularly in rural communities, continue to bear disproportionate burden of hrHPV infection and manifestation to CC and other benign diseases. Multiple factors such as socioeconomic, environmental, and behavioral factors could be associated with CC health disparities. However, emerging research suggests that molecular and biological differences could also play a pivotal role in advanced CC outcomes in racially disparate populations. Periodic screenings through cervical health checkups as well as Pap and HPV-based testing, are necessary in various populations, particularly in rural communities, for CC prevention. At the same time, genetic and epigenetic profiling and an understanding of the molecular genetic basis of CC health in the racial disparity of outcomes is critical to ultimately eradicate CC development and disease-associated burden worldwide and in the USA.
